# Supplementary material for: A retrospective cohort study assessing medication coverage in patients with prostate cancer prescribed luteinizing hormone releasing hormone (LHRH) agonists in England
Source: PLoS One. 2025 Mar 21;20(3):e0315208. doi: 10.1371/journal.pone.0315208 (PMC11927884; doi:10.1371/journal.pone.0315208)
Supplement: Attachment — Submitted filename: PONE-D-24-14050_reviewer.docx [file pone.0315208.s001.docx]

PLOS ONE

A retrospective cohort study assessing medication coverage in patients with prostate cancer prescribed luteinizing hormone releasing hormone (LHRH) agonists in England

--Manuscript Draft--

| Manuscript Number: | PONE-D-24-14050 |
| --- | --- |
| Article Type: | Research Article |
| Full Title: | A retrospective cohort study assessing medication coverage in patients with prostate cancer prescribed luteinizing hormone releasing hormone (LHRH) agonists in England |
| Short Title: | Medication Coverage in Prostate Cancer Patients on LHRH Agonists: A Retrospective Cohort Study in England |
| Corresponding Author: | Ian Sayers  Royal Wolverhampton Hospitals NHS Trust Wolverhampton, UNITED KINGDOM |
| Keywords: | luteinising hormone releasing hormone (LHRH) agonist; triptorelin; goserelin; leuprorelin; patient adherence; prostate cancer |
| Abstract: | Purpose: This study aims to assess adherence to luteinising hormone-releasing hormone (LHRH) agonist treatment for prostate cancer (PC) in England, considering formulation-related differences, their impact on overall survival, and the association with changes in prostate-specific antigen (PSA) levels over time.  Methods: In this retrospective cohort study, utilising primary care data from the Clinical Practice Research Datalink (CPRD) Aurum database linked to Hospital Episode Statistics (HES) and Office for National Statistics (ONS) death registrations, we assessed male patients aged 40 and above diagnosed with PC and prescribed 1-, 3-, or 6-monthly LHRH agonist injections between January 2007 and December 2019. The primary objectives were to measure adherence through proportion of days covered (PDC) and characterize delayed injections, while secondary objectives included assessment of patient demographics, comorbidities, overall survival, and PSA levels. Descriptive statistics were employed, with follow-up restricted to one year for PSA and testosterone measurements due to data availability constraints.  Results: The study included 32,777 patients with PC receiving LHRH agonists. Most patients (67%) were prescribed 3-monthly formulations, while only 2% received 6- monthly formulations. The mean age of the study population was 74.1 years. Over 80% of patients had at least one comorbidity, with hypertension being the most common.  94% of patients initially prescribed the 3-monthly or 6-monthly regimen remained on their original treatment, in contrast to only 38% for the 1-monthly formulation.  Adherence analysis showed that 41.1% of 6-monthly injections were received without delay, compared with 67.9% for the 3-monthly and 77.3% for 1-monthly formulations. A large proportion of patients experienced delays of 14-27 days (32.0%, 33.4%, 54.2%)  and over 27 days (39.6%, 48.3%, 46.6%) across the 1-, 3- and 6-monthly formulations respectively. The mean PDC ranged from 90-91% across the three formulation groups, with 89.9%, 84%, and 88.2% achieving ≥80% adherence for 3-monthly, 1-monthly, and 6-monthly respectively.  Conclusions: This study revealed substantial and consistent dosing delays in LHRH agonist prescriptions across all formulations within primary care settings in England. These delays can negatively affect the control of PC, potentially hindering disease management for affected patients. Future research with a larger population, encompassing a larger cohort using the 6-monthly formulation, is essential for a comprehensive evaluation of the impact of LHRH agonist injection delays on PC progression. |
| Order of Authors: | Mohammad Raja |
|  | Ian Sayers |
|  | Sara Joao Carvalho |
|  | Jennifer Davidson |
|  | Naomi Elster |
|  |  |

|  | Rakesh Heer |
| --- | --- |
|  | Kate Higgs |
|  | Andrew Nolan |
|  | Jelena Sassmann |
| Additional Information: |  |
| Question | Response |
| Financial Disclosure  Enter a financial disclosure statement that describes the sources of funding for the work included in this submission. Review the [submission guidelines](http://journals.plos.org/plosone/s/submission-guidelines#loc-financial-disclosure-statement) for detailed requirements. View published research articles from [*PLOS ONE*](https://journals.plos.org/plosone/) for specific examples.  This statement is required for submission and will appear in the published article if the submission is accepted. Please make sure it is accurate. | The author(s) received no specific funding for this work. |
| Funded studies  Enter a statement with the following details:   - Initials of the authors who received each award - Grant numbers awarded to each author - The full name of each funder - URL of each funder website - Did the sponsors or funders play any role in the study design, data collection and analysis, decision to publish, or preparation of the manuscript? |  |
| Did you receive funding for this work? |  |
| Competing Interests  Use the instructions below to enter a competing interest statement for this submission. On behalf of all authors, disclose any [competing interests](http://journals.plos.org/plosone/s/competing-interests) that could be perceived to bias this work—acknowledging all financial support and any other relevant financial or non- financial competing interests. | Sara Joao Carvalho and Jennifer Davidson are employed by CorEvitas, an organisation that helped to conduct the research. Their participation in this study was supported through funding provided by Ipsen.  Other authors have declared that no competing interests exist. Disclosures have been stated in the manuscript. |

| This statement is required for submission and will appear in the published article if the submission is accepted. Please make sure it is accurate and that any funding sources listed in your Funding Information later in the submission form are also declared in your Financial Disclosure statement.  View published research articles from  [*PLOS ONE*](http://journals.plos.org/plosone/) for specific examples. |  |
| --- | --- |
| NO authors have competing interests  Enter: *The authors have declared that no competing interests exist.*  Authors with competing interests  Enter competing interest details beginning with this statement:  *I have read the journal's policy and the authors of this manuscript have the following competing interests: [insert competing interests here]* |  |
| * typeset |  |
| Ethics Statement  Enter an ethics statement for this submission. This statement is required if the study involved:   - Human participants - Human specimens or tissue - Vertebrate animals or cephalopods - Vertebrate embryos or tissues - Field research   Write "N/A" if the submission does not require an ethics statement.  General guidance is provided below. Consult the [submission guidelines](http://journals.plos.org/plosone/s/submission-guidelines#loc-guidelines-for-specific-study-types) for detailed instructions. Make sure that all information entered here is included in the Methods section of the manuscript. | CPRD has ethics approval from the Health Research Authority to support research using anonymised patient data. Access to a suitable dataset to conduct this study was approved by the then ISAC (now RDG) on 05/11/2021 (protocol number 21_000622). The study adheres to ethical principles as outlined in the Declaration of Helsinki and the guidelines for Good Pharmacoepidemiology Practices from the International Society for Pharmacoepidemiology. It also adhered to all local regulatory requirements applicable to non-interventional studies. |

| Format for specific study types  Human Subject Research (involving human participants and/or tissue)   - Give the name of the institutional review board or ethics committee that approved the study - Include the approval number and/or a statement indicating approval of this research - Indicate the form of consent obtained (written/oral) or the reason that consent was not obtained (e.g. the data were analyzed anonymously)   Animal Research (involving vertebrate animals, embryos or tissues)   - Provide the name of the Institutional Animal Care and Use Committee (IACUC) or other relevant ethics board that reviewed the study protocol, and indicate whether they approved this research or granted a formal waiver of ethical approval - Include an approval number if one was obtained - If the study involved *non-human primates*, add *additional details* about animal welfare and steps taken to ameliorate suffering - If anesthesia, euthanasia, or any kind of animal sacrifice is part of the study, include briefly which substances and/or methods were applied   Field Research  Include the following details if this study involves the collection of plant, animal, or other materials from a natural setting:   - Field permit number - Name of the institution or relevant body that granted permission |  |
| --- | --- |
|  |  |
| Data Availability  Authors are required to make all data underlying the findings described fully available, without restriction, and from the time of publication. PLOS allows rare exceptions to address legal and ethical concerns. See the [PLOS Data Policy](http://journals.plos.org/plosone/s/data-availability) and [FAQ](http://journals.plos.org/plosone/s/data-availability#loc-faqs-for-data-policy) for detailed information. | Yes - all data are fully available without restriction |

| A Data Availability Statement describing where the data can be found is required at submission. Your answers to this question constitute the Data Availability Statement and will be published in the article, if accepted. |  |
| --- | --- |
| Important: Stating ‘data available on request from the author’ is not sufficient. If your data are only available upon request, select ‘No’ for the first question and explain your exceptional situation in the text box. |  |
| Do the authors confirm that all data underlying the findings described in their manuscript are fully available without restriction? |  |
| Describe where the data may be found in full sentences. If you are copying our sample text, replace any instances of XXX with the appropriate details.   - If the data are held or will be held in a public repository, include URLs, accession numbers or DOIs. If this information will only be available after acceptance, indicate this by ticking the box below. For example: *All XXX files are available from the XXX database (accession number(s) XXX, XXX.*). - If the data are all contained within the manuscript and/or Supporting Information files, enter the following: *All relevant data are within the manuscript and its Supporting Information files.* - If neither of these applies but you are able to provide details of access elsewhere, with or without limitations, please do so. For example:   *Data cannot be shared publicly because of [XXX]. Data are available from the XXX Institutional Data Access / Ethics Committee (contact via XXX) for researchers who meet the criteria for access to confidential data.*  *The data underlying the results presented in the study are available* | All relevant data are within the manuscript and its Supporting Information files. |

| *from (include the name of the third party and contact information or URL).*   - This text is appropriate if the data are owned by a third party and authors do not have permission to share the data.   * typeset |  |
| --- | --- |
| Additional data availability information: |  |

<https://www2.cloud.editorialmanager.com/pone/download.aspx?id=36266656&guid=dc69330e-9bed-436a-ad5c-40cf0e4d0889&scheme=1>Manuscript	[Click here to access/download;Manuscript;CPRD Manuscript.docx](https://www2.cloud.editorialmanager.com/pone/download.aspx?id=36266656&guid=dc69330e-9bed-436a-ad5c-40cf0e4d0889&scheme=1)

CPRD Study	CONFIDENTIAL	23 Aug 2023

# 1 A retrospective cohort study assessing medication coverage in patients with prostate cancer

2 **prescribed luteinizing hormone releasing hormone (LHRH) agonists in England**
3 Ian Sayers,^1^ Sara Joao Carvalho,^2^ Jennifer Davidson,^2^ Naomi Elster,^3^ Rakesh Heer,^4^ Mohammad
4 Raja,^5^ Kate Higgs,^5^ Andrew Nolan,^5^ Jelena Sassmann,^5^ 5
6 ^1^Royal Wolverhampton Hospitals NHS Trust, UK
7 ^2^CorEvitas, UK
8 ^3^Prostate Cancer Research, UK
9 ^4^Imperial College London, UK
10 ^5^Ipsen, UK
11 *Corresponding author: Dr Ian Sayers
12 Email: [igsayers8@outlook.com](mailto:igsayers8@outlook.com)

13

| Author | ORCID |
| --- | --- |
| Dr Ian Sayers | 0000-0002-7685-178X |
| Sara Joao Carvalho | 0000-0002-5512-4540 |
| Jennifer Davidson | 0000-0003-1599-7504 |
| Naomi Elster | [0000-0003-3865-5662](https://orcid.org/0000-0003-3865-5662) |
| Rakesh Heer | [0000-0003-1952-7462](https://orcid.org/0000-0003-1952-7462) |
| Mohammad Raja | 0009-0001-8873-7968 |
| Kate Higgs |  |

| Andrew Nolan | 0000-0003-0799-2914 |
| --- | --- |
| Jelena Sassmann |  |

14

15 **Target journal:** *PLOS ONE*
16 **Short title:** CPRD study
17 **Keywords:** luteinising hormone releasing hormone (LHRH) agonist; triptorelin; goserelin;
18 leuprorelin; patient adherence; prostate cancer
19 **Word count:** 3,611
20 **Figures:** 5 ; **Tables:** 3
21 **Supplementary figures:** 0; **Supplementary tables:** 0
22 **References:** 11
23 **Statements and declarations**
24 Sara Joao Carvalho and Jennifer Davidson are employed by CorEvitas, an organisation that
25 helped to conduct the research. Their participation in this study was supported through funding
26 provided by Ipsen.

# 27 Disclosures

28 Ian Sayers – No disclosures
29 Sara Joao Carvalho – No disclosures
30 Jennifer Davidson – No disclosures
31 Naomi Elster – No disclosures
32 Rakesh Heer – No disclosures
33 Jelena Sassmann – Ipsen employee and shareholder
34 Kate Higgs - Ipsen employee
35 Andrew Nolan - Ipsen employee
36 Mohammad Raja – Ipsen employee

# 37 Author Contributions

38 Substantial contributions to study conception/design, or acquisition/analysis/interpretation of
39 data: all authors; drafting of the publication or revising it critically for important intellectual
40 content: all authors; final approval of the publication: all authors.

# 41 Funding

42 This study was sponsored and funded by Ipsen, UK. The sponsor was involved in the design of
43 the study, analysis and interpretation of the data, and development of the manuscript.

# 44 Data Sharing Statement

45 This study was conducted using data obtained under licence from the United Kingdom
46 Medicines and Healthcare products Regulatory Agency (MHRA) following study approval by the
47 Independent Scientific Advisory Committee (ISAC). The Clinical Practice Research Datalink
48 (CPRD) Aurum dataset is sourced from GP practices using EMIS® GP software. Data from the
49 Hospital Episode Statistics (HES)® dataset were linked to CPRD Aurum data by NHS Digital and
50 are re-used with the permission of The Health & Social Care Information Centre (HSCIC). All
51 rights reserved. Therefore, the data cannot be broadly disclosed or made publicly available.
52 Access to CPRD and linked datasets can be requested by application via the CPRD Research
53 Data Governance (RDG) process (replacement of ISAC). The interpretation and conclusions
54 contained in this study are those of the author/s alone.

# 55 Ethics approval

56 CPRD has ethics approval from the Health Research Authority to support research using
57 anonymised patient data. Access to a suitable dataset to conduct this study was approved by
58 the then ISAC (now RDG) on 05/11/2021 (protocol number 21_000622). The study adheres to
59 ethical principles as outlined in the Declaration of Helsinki and the guidelines for Good
60 Pharmacoepidemiology Practices from the International Society for Pharmacoepidemiology. It
61 also adhered to all local regulatory requirements applicable to non-interventional studies.

62

# 63 Abstract

64 **Purpose:** This study aims to assess adherence to luteinising hormone-releasing hormone (LHRH)
65 agonist treatment for prostate cancer (PC) in England, considering formulation-related
66 differences, their impact on overall survival, and the association with changes in prostate-
67 specific antigen (PSA) levels over time.
68 **Methods:** In this retrospective cohort study, utilising primary care data from the Clinical
69 Practice Research Datalink (CPRD) Aurum database linked to Hospital Episode Statistics (HES)
70 and Office for National Statistics (ONS) death registrations, we assessed male patients aged 40
71 and above diagnosed with PC and prescribed 1-, 3-, or 6-monthly LHRH agonist injections
72 between January 2007 and December 2019. The primary objectives were to measure
73 adherence through proportion of days covered (PDC) and characterize delayed injections, while
74 secondary objectives included assessment of patient demographics, comorbidities, overall
75 survival, and PSA levels. Descriptive statistics were employed, with follow-up restricted to one
76 year for PSA and testosterone measurements due to data availability constraints.
77 **Results:** The study included 32,777 patients with PC receiving LHRH agonists. Most patients
78 (67%) were prescribed 3-monthly formulations, while only 2% received 6-monthly formulations.
79 The mean age of the study population was 74.1 years. Over 80% of patients had at least one
80 comorbidity, with hypertension being the most common. 94% of patients initially prescribed
81 the 3-monthly or 6-monthly regimen remained on their original treatment, in contrast to only
82 38% for the 1-monthly formulation. Adherence analysis showed that 41.1% of 6-monthly
83 injections were received without delay, compared with 67.9% for the 3-monthly and 77.3% for
84 1-monthly formulations. A large proportion of patients experienced delays of 14-27 days
85 (32.0%, 33.4%, 54.2%) and over 27 days (39.6%, 48.3%, 46.6%) across the 1-, 3- and 6-monthly
86 formulations respectively. The mean PDC ranged from 90-91% across the three formulation
87 groups, with 89.9%, 84%, and 88.2% achieving ≥80% adherence for 3-monthly, 1-monthly, and
88 6-monthly respectively.
89 **Conclusions:** This study revealed substantial and consistent dosing delays in LHRH agonist
90 prescriptions across all formulations within primary care settings in England. These delays can
91 negatively affect the control of PC, potentially hindering disease management for affected
92 patients. Future research with a larger population, encompassing a larger cohort using the 6-
93 monthly formulation, is essential for a comprehensive evaluation of the impact of LHRH agonist
94 injection delays on PC progression.

95

# 96 Plain language summary

97 Patients with prostate cancer (PC) are commonly treated with medicines called luteinising
98 hormone-releasing hormone (LHRH) agonists. These medications, available in formulations
99 administered at different intervals (1-, 3- and 6-monthly), play a crucial role in the management
100 of PC. This study aimed to investigate how closely men in England with PC followed their LHRH
101 agonist treatment plan and if treatment delays varied between patients receiving their LHRH
102 agonist injections at different time intervals (1-, 3- and 6-monthly), and if these delays could
103 affect their treatment outcomes. We looked at the records of 32,777 patients. The study
104 included male patients aged 40 years and above who had PC and were prescribed 1-, 3-, or 6-
105 monthly LHRH agonist injections during the period from January 2007 to December 2019. These
106 records were obtained from different datasets in both primary care (general practitioner /
107 family doctor) and secondary care (hospitals). Most of the patients (67%) were administered 3-
108 monthly LHRH agonist injections for their PC, whereas only a small number (2%) received the 6-
109 monthly formulation. Delays were common across all injection schedules, with a large
110 proportion of patients (45.5%) experiencing treatment delays of over 27 days, which could
111 reduce the effectiveness of LHRH agonist treatment for PC. Patients on the three different
112 formulations (1-, 3- and 6-monthly) demonstrated similar levels of treatment plan compliance.
113 Further research with a larger group of patients, particularly a greater number of patients on
114 the 6-monthly LHRH agonist injection schedule, is needed to fully understand the impact of

115

116

treatment delays on outcomes in patients with PC.

# 117 Introduction

118 Prostate cancer (PC) is the most frequently diagnosed cancer in men and is the second leading
119 cause of male cancer deaths in England (1). A mainstay of treatment for locally advanced and
120 metastatic PC is androgen-deprivation therapy (ADT), specifically luteinising hormone-releasing
121 hormone (LHRH) agonists which lower the level of testosterone (2). LHRH agonists inhibit
122 luteinising hormone secretion by inducing prolonged activation of LHRH receptors leading to
123 desensitisation. This reduces testosterone production, which slows the growth and progression
124 of PC cells (3). ADT is also used in intermediate and high-risk localised PC, in the neoadjuvant
125 and adjuvant setting prior to and after radical therapy is initiated, and as an adjuvant treatment
126 to radical prostatectomy in patients with locally advanced PC at high risk of disease progression
127 (4). Castrate levels were previously described as <50ng/dL, however a level of <20 ng/dL is now
128 recommended (5) and delays in LHRH agonist dosing may lead to an increase from these

129

130

recommended castration levels of testosterone.

131 In England, most patients receive their first LHRH agonist injection in the secondary care setting
132 and are then further treated in primary care. The routine receipt of LHRH agonist injections can
133 be complex in the community setting, often consisting of multiple steps such as: decision to
134 initiate in secondary care, communication to primary care, patient booking injection
135 appointments, requesting the prescription, collecting their prescription from a community
136 pharmacy, and taking the prescribed injection with them to their appointment at a primary care
137 centre for administration. The standard of care and level of patients’ involvement, as well as

138

139

the steps that must be completed to receive an injection vary across England.

140 LHRH agonists used in PC treatment are commercially available in the UK as 1-, 3- or 6-monthly
141 injection formulations. Some LHRH agonists are also available as every 28-day formulations,
142 which will be referred to as 1-monthly in this study for simplicity. A study analysing data from
143 the USA investigated the timeliness of LHRH agonist dosing by the different formulations (1, 3, 4
144 and 6-monthly) (6). Results from the analysis suggested late injections, defined as receipt on
145 any day after the pre-specified 28-, 84-, 112- or 168-day window, were common, with 84%
146 (71,426) of injections received late. When an extended definition was used, defined as delay of
147 four or more days after the pre-specified 28-, 84-, 112- or 168-day window, late injections were
148 less frequent, but more than one quarter (27%) (22,959) of injections were still received late
149 (6). With a lack of existing UK-specific data, our study assessed adherence to LHRH agonist
150 treatment in England. We examined formulation-related adherence differences and its effect
151 on overall survival. We also analysed how different formulations and adherence rates affected
152 prostate specific antigen (PSA) changes over time. It is important to note that while this analysis
153 assumes a likely causal relationship between variance in PSA and adherence schedule, such a

154

155

connection may not always be definitive.

# 156 Methods

157 Our retrospective cohort study used longitudinal primary care data drawn from the clinical
158 management system used in GP practices and collated by the CPRD in a database named
159 Aurum. The CPRD Aurum data is linked to secondary care data in the form of a reimbursement
160 dataset called the Hospital Episode Statistics (HES) and death registrations collated by the
161 Office for National Statistics (ONS). This study utilised anonymised structured data, preventing
162 the authors with data access from identifying individual participants both during and after data
163 collection.
164 Male patients aged 40 years and above, diagnosed with PC between 01 January 2007 and 31
165 December 2019 (study period [duration during which data was collected and analysed]: 01 April
166 2007 – 31 March 2020, index period [time in which patients received treatment]: 01 January
167 2007 – 31 December 2019) who had been prescribed an LHRH agonist injection, available in 1-,
168 3-, or 6-monthly formulations licensed in the UK within the primary care setting were included
169 (Figure 1). The cut-off date for the inclusion criteria was set to prevent the effects of a change
170 in the standard of care during the COVID-19 pandemic affecting the analysis. A minimum of two
171 primary care prescriptions were required to allow the calculation of the proportion of days
172 covered (PDC). Patients were stratified by LHRH agonist formulation. Additionally, patients
173 were further stratified if they remained on their initial formulation or switched to another
174 formulation: from 1-monthly to 3- or 6-monthly, from 3-monthly to 1- or 6-monthly, and from
175 6-monthly to 1- or 3-monthly. Follow-up was conducted until the earliest occurrence of death,
176 loss to follow-up in the dataset (i.e., last data collection for the primary care practice or patient

177

178

transferred out of the practice) or the end of the index period.

179 The primary objectives of this study were to measure adherence to the 1-, 3- and 6-monthly
180 LHRH agonist injection formulations through PDC, and to describe the number of delayed
181 injections occurring <4, 4-6, 7-13, 14-27 and ≥28 days beyond the pre-specified time window as
182 per the summary of product characteristics for each of the 1-, 3- and 6-month formulations.
183 The number of delayed injections stratified for the intervals <4, 4-6, 7-13, 14-27 and ≥28 days
184 are presented with absolute (and percentage) number of counts. Injections occurring with a
185 delay of <4 days were classified as “no delay”. PDC was calculated by dividing the number of
186 days between the date of receiving LHRH agonist injection to the subsequent LHRH agonist
187 injection, by the number of days the LHRH agonist injection was intended to cover. When
188 calculating PDC, it was assumed that prescriptions were for 28 days x the number of months
189 the regimen is intended to cover + 3 days to allow for delays caused by weekends and public
190 holidays. Prescription issue dates served as a proxy for adherence schedule, as the actual
191 administration dates of LHRH agonists were not available in the CPRD dataset. This method is
192 indicative of actual administration dates, although potentially underestimates delays in some

193

194

patients.

195 A secondary objective of this study was to characterise patients by demographics,
196 comorbidities, and time from diagnosis by LHRH formulation. Comorbid conditions at baseline
197 were summarised using the Charlson Comorbidity Index (CCI) score and for specific predefined
198 conditions: hypertension, high low-density lipoprotein (LDL), diabetes, obesity, atrial fibrillation,
199 acute myocardial infarction, ischemic stroke, and heart failure. Description of the cause of
200 death and overall survival for patients treated with LHRH agonist injections stratified by LHRH
201 formulation was another secondary objective of this study. Kaplan Meier curves were
202 generated to describe the median survival and survival probability for all patients and
203 increasing PDC intervals <80%, 80-<85%, 85-<90%, 90-<95%, 95-<100% and 100% were
204 reported from 6 to 48 months at every 6-month interval. The final secondary objective of this
205 study was to compare and describe PSA levels in patients that made up the study population
206 stratified by formulation prescribed and adherence measured by PDC. Follow-up was restricted
207 to one year, due to a lack of data availability.

208

209 Exploratory endpoints regarding PSA and testosterone distribution have been reported. Less
210 than 6% of the whole cohort had a testosterone measurement recorded in CPRD dataset in the
211 first year of follow up. Beyond the first year, the data became even more sparse and due to this
212 missing data, no further analysis was conducted. Almost 50% of the whole cohort had at least
213 one PSA measurement available in the CPRD dataset during follow up. For similar reasons as to
214 testosterone distribution, we did not report past 1 year of follow-up due to the large volume of

215

216

missing data.

217 The statistical analysis was descriptive therefore no formal statistical significance testing was
218 performed. Descriptive statistics were presented as: number of available observations (N),
219 number of missing values (missing), mean, SD, median, 1st and 3rd quartile and the range
220 (minimum, maximum) for continuous variables; the absolute and relative (percentage) numbers

221

222

presented for categorical and discrete variables.

# 223 Results

224 *Demographic characteristics*
225 The study population comprised of 32,777 patients with PC treated with an LHRH agonist with
226 at least 2 injections recorded during follow-up in primary care (Figure 2). Approximately 67%
227 (21,910) of patients were initially prescribed the 3-monthly LHRH agonist injection, while 32%
228 (10,365) were initially prescribed the 1-monthly formulation, and only 2% (502) received the 6-
229 monthly formulation initially (Figure 2).

230

231 The overall mean age was 74.1 years (SD 8.7), with minor variations by formulation: patients on
232 the 1-monthly, 3-monthly, and 6-monthly treatments had mean ages of 72.7 years (SD 8.3),
233 74.7 years (SD 8.8), and 74.5 years (SD 8.8), respectively (Table 1). Over 80% of patients had at
234 least one comorbidity (based on number of patients with a record of comorbidities), with
235 diagnosed hypertension being the most common (around 60% across all cohorts) (Table 1).
236 Using the CCI, most patients (~40%) had scores of 1 or 2. Higher CCI scores were generally
237 observed in patients on longer formulations (≥3: 9.5% for 1-monthly formulation versus 13.6%

238

239

for 6-monthly formulation) (Table 1).

240 Patients initially treated with the 3-monthly regimen were least likely to switch to a different
241 formulation (1-monthly or 6-monthly), with 94% (20,687) remaining on their original treatment.
242 Similarly, 94% (474) of patients on the 6-monthly regimen also did not switch treatments,
243 although the cohort size was small in the time period covered. In comparison, only 38% (3,891)
244 of patients on the 1-monthly treatment stayed unchanged. Most patients (20% [6,447/32,777])
245 were switched from their initially prescribed formulation to the 3-monthly formulation rather

246

247

than the 1-monthly (3% [839/32,777]) or 6-monthly (1% [464/32,777]).

248 The overall mean follow-up period for the whole study population was 54.3 (≈ 4.5 years)
249 months, SD 37.2. Patients treated with the 6-monthly formulation had a shorter follow-up
250 period (mean of 37.5 months, SD 18.1) in keeping with when this preparation came into use. In
251 contrast, longer follow-ups were observed for the 1-monthly formulation (mean of 63.1
252 months, SD 40.4) and the 3-monthly formulation (mean of 50.5 months, SD 35.2). The
253 predominant ethnicity within the study population was white, constituting 81.5% of the overall

254

255

participants.

256 Based on calculations using ONS data (Table 2), the prevalence of black or black British patients
257 in the study population (0.40% [1,175]) was nearly twice that of white patients (0.24%
258 [26,720]). This finding aligns with existing research indicating that black men have a higher
259 likelihood of developing prostate cancer compared with individuals from other racial

260

261

backgrounds (7).

262 *Primary endpoint - patient adherence measured by delayed LHRH agonist injections*
263 Data presented show that the proportion of injections received without a delay (within 4 days
264 of date receipt was due) was 41.1% for 6-monthly, 67.9% for the 3-monthly and 77.3% for 1-
265 monthly LHRH agonist formulation (Figure 3). However, a large proportion of patients
266 experienced at least one injection delay of 14-27 days (32.0% for 1-monthly, 33.4% for the 3-
267 monthly and 54.2% for 6-monthly LHRH agonist formulation) (Figure 3). Furthermore, 39.6%,
268 48.3% and 46.6% of patients in the 1-, 3-, and 6-monthly LHRH agonist formulation cohorts

269

270

experienced at least one injection delay of over 27 days (Figure 3).

271 *Primary endpoint - patient adherence measured by proportion of days covered (PDC)*
272 The mean PDC was similar across all formulation groups. Both the 3- and 6-monthly cohorts
273 achieved a PDC of 91% and the 1-monthly cohort had a PDC of 90% (Table 3). The 1-monthly
274 cohort contained 18.5% (1,919) of patients who achieved a 100% PDC, whereas among the 3-
275 and 6-monthly cohorts this percentage decreased to 11.6% (2,537) and 6% (30), respectively
276 (Table 3). When using a PDC of ≥80% as a cut-off to define adherence, 89.9% of the 6-monthly
277 cohort were adherent versus 84% and 88.2% for the 1- and 3-monthly cohorts, respectively.
278 Table 3 contains additional PDC interval measurements for the 1-, 3-, and 6-monthly

279

280

formulations.

281 *Secondary endpoint - overall survival analysis*
282 The (Figure 4) median overall survival (OS) was 111 months (95% CI = 107-114 months) for the
283 1-monthly formulation, 72 months (95% CI = 71-74 months) for the 3-monthly and 58 months
284 (95% CI = 55-66 months) for the 6-monthly (Figure 4). It should be noted that a large proportion
285 (62%) of patients initially commenced on a 1-monthly formulation were then subsequently
286 switched to a longer duration formulation, so the data represents mixed dose regimens. When
287 stratifying the Kaplan Meier curves by PDC, a survival probability of at least 60% is observed for
288 the 1- and 3-monthly formulations at 48 months regardless of PDC. Furthermore, the analysis
289 revealed that within the 1-monthly cohort, patients who achieved a 100% PDC demonstrated
290 an increased survival probability (Figure 4). Due to the small sample size in the 6-monthly

291

292

cohort, there was no stratification in survival by PDC level.

293 *Secondary endpoint - cause of death*
294 Among the patients who died during follow-up, PC was considered the highest primary (69.6%)
295 and secondary (16.0%) cause of death (Figure 5, Figure 6). Prostate cancer being recorded as a
296 secondary cause of death is in accordance with the standard practice on UK death certificates.
297 In such cases, the primary cause of death is the condition that directly leads to the fatality,
298 while the secondary cause may represent an underlying or contributing factor.

# 299 Discussion

300 Most patients with PC in the study were initially started on a 3-monthly LHRH agonist
301 formulation. Patients switching formulations mostly switched from 1-monthly to 3-monthly.
302 The vast majority (94%) of patients initially prescribed a 3-monthly or 6-monthly formulation
303 did not switch, which suggests that these formulations are well-tolerated and practical for
304 healthcare services to deliver. Formulation usage and patient characteristics were broadly

305

306

consistent with data from the USA (6).

307 A very high proportion of patients were exposed to delays (defined as a delay greater than or
308 equal to 4 days) in the administration of their LHRH agonist injections, across all formulation
309 cohorts. Overall, 45.5% (14,925) of patients in the study population experienced at least one
310 injection delay exceeding 27 days, more specifically, 39.6% (4,106), 48.3% (10,585), and 46.6%
311 (234) of patients in the 1-monthly, 3-monthly, and 6-monthly cohorts, respectively. These
312 delays may reasonably be expected to result in a reduction in testosterone suppression in some
313 cases. While the absence of testosterone measurements in the CPRD database means we have
314 not been able to directly confirm this, a potential for avoidable disease progression seems to
315 exist. In addition, the methodology used is likely to have resulted in a conservative estimate of

316

317

treatment delays because it is based on the point of prescription rather than administration.

318 A possible explanation for delays in the 3- and 6-monthly cohorts could be that prescriptions for
319 LHRH agonist injections are based on calendar time rather than the intervals recommended by
320 manufacturers. This deviation may be influenced by factors such as the limited availability of
321 healthcare professionals, including general practitioners (GPs) and nurses. This would not have
322 affected delays in the 1-monthly formulation cohort as, delays of ‘less than 4 days’ were not
323 classified as a ‘delay’ in this study. Primary care practices determine how far in advance
324 appointments can be made and scheduling appointments six months ahead is logistically
325 difficult. Furthermore, based on our own clinical practice experiences, there may be a
326 perception that the clinical consequences are less significant for treatment delays with the
327 longer-acting formulations as the medicine and its effects are perceived to persist in the system
328 for longer. The study results align with a similar analysis in the USA (6), which found more
329 instances of late dosing for longer-acting formulations (6-monthly: 95% late, 1-monthly: 73%

330

331

late).

332 Overall, findings in relation to the 6-monthly formulation should be interpreted with caution
333 due to the small number of patients who received this formulation. The relatively low adoption
334 of the 6-monthly formulation can be attributed to its delayed availability for use in the NHS
335 compared with the other formulations (although contemporary practice has evolved since the
336 COVID-19 pandemic) (8). Adherence rates were similar across formulations, with some minor
337 differences for those on a 6-monthly formulation. A lower proportion of patients initiated on a
338 6-monthly formulation achieved a 100% treatment coverage when compared with those
339 initiated on 1- and 3-monthly formulations. However, among patients in the 6-monthly group,
340 89.9% achieved a PDC of ≥80%, compared with 84% and 88.2% for the 1- and 3-monthly groups
341 respectively, which shows that these patients achieved the highest levels of adherence to their
342 prescribed therapy plan.
343 It is important to note that the data available for testosterone and PSA measurements were
344 limited, with less than 6% of individuals having testosterone measurements recorded in primary
345 care, and only 50% having at least one PSA measurement during follow-up. PSA and
346 testosterone measurements are crucial for monitoring and managing PC, and the lack of
347 recorded data on these measurements poses a significant challenge. One possible explanation
348 for the low recorded testosterone and PSA measurements in the study population is that data
349 from CPRD is limited to primary care, and that these data are only available in secondary care
350 electronic health records where prostate cancer is typically diagnosed and managed.
351 Additionally, it is worth noting that this data is not available in the HES database, which does
352 contain secondary care data. However, the HES database is more focused on patient outcomes
353 for reimbursement purposes rather than laboratory results, and consequently lacks information
354 on testosterone and PSA measurements. Another explanation may be attributed to factors such
355 as the cost of testing or a potential lack of clinician awareness regarding the necessity to assess

356

357

these metrics.

358 The primary consideration when evaluating the overall survival is the limited sample size and
359 shorter follow-up period in the 6-monthly cohort. When comparing patients on the 6- and 3-
360 monthly formulation, there was no statistically significant difference in overall survival. It
361 should also be noted that these are ‘all cause’ deaths and not PC specific, of which there was a
362 lower proportion in the 6-monthly formulation cohort. Additionally, competing risks have not
363 been accounted for. The study found statistically significant differences in overall survival (OS)
364 between the 1-monthly and 3-monthly formulations, with patients receiving the 1-monthly
365 formulation exhibiting a higher median OS than those receiving the 3-monthly formulation.
366 However, it's important to note that many patients initially started on the 1-monthly
367 formulation were later switched to longer-duration formulations. Based on our experience, this
368 change is primarily due to hospital discharge policies, which mandate a 7-day medicines supply
369 upon discharge to lower treatment costs incurred by the hospital. Since the smallest and
370 cheapest pack size available is the 1-monthly formulation, follow-up care is often transferred to
371 primary care where GPs, considering patient preferences and their workload, may choose to
372 prescribe 3-month or 6-month formulations. Upon stratifying the data by PDC, those patients
373 receiving the 1-monthly formulation, who exhibited a 100% coverage rate, displayed an
374 increased probability of survival (after approximately 30 months). However, it is important to
375 highlight that a significant portion of patients who were initially started on the 1-monthly
376 formulation later switched to a longer duration regimen, resulting in a dataset that contains
377 mixed dosing regimens. Studies have indicated that discontinuing lower duration LHRH agonist
378 injections could lead to a quicker regeneration of testosterone levels compared with when
379 stopping longer duration formulations (9). This finding offers a potential explanation for fewer
380 treatment delays and the increased sensitivity of patients on the 1-monthly formulation to

381

382

treatment delays.

383 In line with previous research (10), this study, along with data from the Office for National
384 Statistics (ONS), demonstrates that the prevalence of PC among patients from a black or black
385 British background was approximately twice as high compared with white patients,
386 emphasizing findings across multiple studies and geographies.
387 *Limitations*
388 A significant limitation of this study pertains to the assessment of the 6-monthly LHRH agonist
389 formulation. Unlike the other formulations examined, the 6-monthly formulation product had a
390 relatively shorter period of formulary availability within the UK based NHS during the study
391 period (11). Consequently, the sample size of patients receiving the 6-monthly formulation was
392 notably smaller in comparison to the 1-monthly and 3-monthly cohorts. This lack of data limits
393 our ability to draw valid conclusions regarding the long-term treatment outcomes and overall

394

395

survival of patients on the 6-monthly formulation compared with the other formulations.

396 Another limitation is that the “dates” captured in the CPRD database relate to the date the
397 prescription was issued, rather than the administration of injection itself. As such, the
398 administration date was inferred from the prescription issue date. As prescriptions can only be
399 administered after issue and collection, this would mean that the dosing delays could be
400 significantly underestimated in this analysis but cannot be shorter. Furthermore, it is important
401 to note that the CPRD database does not provide reasons behind the delays, which is an
402 additional limitation of this study. Without access to the specific reasons for these delays, the
403 analysis is limited in its ability to capture the full spectrum of factors influencing treatment
404 adherence.

405

406 In addition, a significant number of patients will have received their first prescription in
407 secondary care, and this cannot be captured in the available CPRD-HES linked database as the
408 secondary care component only covers medical activity and does not contain secondary care
409 prescription data. The impact of this would mean that for any patients first prescribed a LHRH
410 agonist in secondary care, the first prescription captured in the primary care is not their
411 genuine treatment start. Although this could account for a higher rate of switching with primary

412

413

care prescribers preferring a longer interval to reduce the number of clinic appointments.

414	Neither the administration of other treatments, PC-related surgery, nor the time from diagnosis

415	to treatment were considered in this analysis. There may be imbalance between locally

416	advanced and metastatic disease across the formulation groups which cannot be distinguished

417

418

in the data.

419 *Concluding statements*
420 Overall, the study identified significant and systemic dosing delays across all formulations of
421 LHRH agonist prescriptions in primary care settings across England, these have the potential to
422 significantly adversely affect patients with PC. Further studies with a larger population,
423 including a larger cohort receiving the 6-monthly formulation will be necessary to accurately

424

425

assess the impact of delays on PC progression.

# 427 Acknowledgements

428	We would like to express our gratitude to Ravina Barrett for her assistance in reviewing this

429	paper. Her expertise and constructive criticism have been instrumental in helping create the

430

431

432

433

434

435

436

437

438

439

440

441

442

443

444

445

446

447

final version of this paper.

449	**References**

450	1. Bycroft C, et al. The UK Biobank resource with deep phenotyping and genomic data. Nature.

451	2018 Oct;562:203-209. doi: 10.1038/s41586-018-0579-z.

452 2. EAU Guidelines Prostate Cancer - TREATMENT - Uroweb. [Internet]. [cited 2023 Jul 1].
453 Available from: <https://uroweb.org/guidelines/prostate-cancer/chapter/treatment>.
454 3. Ortmann O, Weiss JM, Diedrich K. Gonadotrophin-releasing hormone (GnRH) and GnRH
455 agonists: mechanisms of action. Reproductive BioMedicine Online. 2002;5:1–7. doi:
456 10.1016/S1472-6483(11)60210-1.
457 4. Sharifi N, Gulley JL, Dahut WL. Androgen Deprivation Therapy for Prostate Cancer. JAMA.

458	2005 Jul;294(2):238–244. doi: 10.1001/jama.294.2.238.

459 5. EAU - EANM - ESTRO - ESUR - ISUP - SIOG Guidelines on Prostate Cancer. [cited 2023 Sep
460 27]. Available from: [https://d56bochluxqnz.cloudfront.net/EAU-EANM-ESTRO-ESUR-ISUP-](https://d56bochluxqnz.cloudfront.net/EAU-EANM-ESTRO-ESUR-ISUP-SIOG-Guidelines-on-Prostate-Cancer-2023_2023-06-13-141145.pdf)
461 [SIOG-Guidelines-on-Prostate-Cancer-2023_2023-06-13-141145.pdf](https://d56bochluxqnz.cloudfront.net/EAU-EANM-ESTRO-ESUR-ISUP-SIOG-Guidelines-on-Prostate-Cancer-2023_2023-06-13-141145.pdf).
462 6. David CE, et al. The Impact of Late Luteinizing Hormone-Releasing Hormone Agonist Dosing
463 on Testosterone Suppression in Patients with Prostate Cancer: An Analysis of United States
464 Clinical Data. J Urol. 2020 Apr;203(4):743–750. doi: 10.1097/JU.0000000000000577.
465 7. Ben-Shlomo Y, et al. The Risk of Prostate Cancer amongst Black Men in the United Kingdom:
466 The PROCESS Cohort Study. Eur Urol. 2008;53(1):99-105. doi:
467 10.1016/j.eururo.2007.02.047.
468 8. Barrett R, Barrett R, Dhar K, Birch B. Gonadorelins adherence in Prostate cancer: A Time‐
469 series analysis of England’s national prescriptions during the COVID‐19 pandemic (from Jan
470 2019 to Oct 2020). BJUI Compass. 2021;2(6):419–427. doi: 10.1002/bco2.101.
471 9. Pai H, Pickles T, Keyes M, Jones SE, McDonald R, Lesperance M, Berthelet E. Randomized
472 study evaluating testosterone recovery using short-versus long-acting luteinizing hormone
473 releasing hormone agonists. Can Urol Assoc J. 2011;5(3):173–179. doi: 10.5489/cuaj.10102.
474 10. Hinata N, Fujisawa M. Racial differences in prostate cancer characteristics and cancer-
475 specific mortality: An overview. World J Men’s Health. 2022;40(2):217. doi:
476 10.5534/wjmh.210070.
477 11. Barrett R, Birch B. Triptorelin therapy for lower urinary tract symptoms (LUTS) in prostate
478 cancer patients: A systematic meta‐analysis. BJUI Compass. 2023. doi: 10.1002/bco2.292.

Tables and Figures	[Click here to access/download;Figure;CPRD Manuscript](https://www2.cloud.editorialmanager.com/pone/download.aspx?id=35960434&guid=814cf040-0b4d-4245-8038-1886aed8ec7a&scheme=1)[<https://www2.cloud.editorialmanager.com/pone/download.aspx?id=35960434&guid=814cf040-0b4d-4245-8038-1886aed8ec7a&scheme=1> Tables and Figures.docx](https://www2.cloud.editorialmanager.com/pone/download.aspx?id=35960434&guid=814cf040-0b4d-4245-8038-1886aed8ec7a&scheme=1)

1	**Table 1. Baseline characteristics table**

| Metrics | | Whole study population | 1-month LHRH agonist injection | 3-month LHRH agonist injection | 6-month LHRH agonist injection |
| --- | --- | --- | --- | --- | --- |
| Total number of patients | | 32,777 | 10,365 | 21,910 | 502 |
| Total time in cohort (patient days) | | 53,371,173 | 19,621,778 | 33,184,728 | 564,667 |
| Follow up  time (months)  * | Mean | 54.3 | 63.1 | 50.5 | 37.5 |
|  | Standard deviation | 37.2 | 40.4 | 35.2 | 18.1 |
|  | Median | 45.5 | 57.1 | 41.3 | 36.0 |
|  | Maximum | 158.1 | 157.4 | 158.1 | 104.0 |
|  | Minimum | 6.0 | 6.0 | 6.0 | 6.8 |
|  | Q1-Q3 | 22.8 – 79.07 | 27.7 – 94.5 | 21.37 – 72.37 | 22.8 – 49.62 |
| Age (years) | Mean | 74.1 | 72.7 | 74.7 | 74.5 |
|  | Standard deviation | 8.7 | 8.3 | 8.8 | 8.8 |
|  | Median | 74.0 | 73.0 | 75.0 | 75.0 |
|  | Maximum | 100 | 100 | 100 | 97 |
|  | Minimum | 40 | 40 | 41 | 48 |
|  | Q1-Q3 | 68 to 80 | 67 to 78 | 69 to 81 | 68 to 81 |
| Age groups | <65 years | 4431 (13.5%) | 1659 (16%) | 2710 (12.4%) | 62 (12.4%) |
|  | 65-69 years | 5195 (15.9%) | 1874 (18.1%) | 3239 (14.8%) | 82 (16.3%) |
|  | 70-74 years | 7141 (21.8%) | 2520 (24.3%) | 4521 (20.6%) | 100 (19.9%) |
|  | 75-79 years | 7307 (22.3%) | 2300 (22.2%) | 4897 (22.4%) | 110 (21.9%) |
|  | ≥80 years | 8703 (26.6%) | 2012 (19.4%) | 6543 (29.9%) | 148 (29.5%) |
| Charlson comorbidity index score | Number of patients with available data** | 16829 (51.3%) | 4962 (47.9%) | 11601 (53%) | 266 (53%) |
|  | 0** | 0 (0%) | 0 (0%) | 0 (0%) | 0 (0%) |
|  | 1-2 | 13230 (40.4%) | 3981 (38.4%) | 9051 (41.3%) | 198 (39.4%) |
|  | 3-4 | 2916 (8.9%) | 806 (7.8%) | 2058 (9.4%) | 52 (10.4%) |

|  | 5-9 | 678 (2.1%) | 174 (1.7%) | 489 (2.2%) | 15 (3%) |
| --- | --- | --- | --- | --- | --- |
|  | 10-14 | 5 (0%) | <5† | <5† | <5† |
|  | 15+ | 0 (0%) | 0 (0%) | 0 (0%) | 0 (0%) |
|  | Number of patients without  available data | 15948 (48.7%) | 5403 (52.1%) | 10309  (47.1%) | 236 (47%) |
| Comorbidities | Number of patients with record of  comorbidities** | 27091 (82.7%) | 8571 (82.7%) | 18102  (82.6%) | 418 (83.3%) |
|  | Diagnosed hypertension | 19438 (59.3%) | 5957 (57.5%) | 13170  (60.1%) | 311 (62%) |
|  | High LDL | 17080 (52.1%) | 5732 (55.3%) | 11089  (50.6%) | 259 (51.6%) |
|  | Diabetes | 6041 (18.4%) | 1784 (17.3%) | 4149 (18.9%) | 98 (19.5%) |
|  | Obesity | 6993 (21.3%) | 2183 (21.1%) | 4706 (21.5%) | 104 (20.7%) |
|  | Atrial fibrillation | 4015 (12.3%) | 1082 (10.4%) | 2878 (13.1%) | 55 (11%) |
|  | Acute myocardial infarction | 2791 (8.5%) | 796 (7.7%) | 1958 (8.9%) | 37 (7.4%) |
|  | Ischemic stroke | 953 (2.9%) | 241 (2.3%) | 705 (3.2%) | 7 (1.4%) |
|  | Heart failure | 1770 (5.4%) | 459 (4.4%) | 1279 (5.8%) | 32 (6.4%) |
|  | Number of patients with no record of comorbidities | 5686 (17.4%) | 1794 (17.3%) | 3808 (17.4%) | 84 (16.7%) |
| Geographical region | East of England | 1769 (5.4%) | 402 (3.9%) | 1256 (5.7%) | 111 (22.1%) |
|  | South East | 3011 (9.2%) | 1350 (13%) | 1657 (7.6%) | <5† |
|  | London | 4676 (14.3%) | 2044 (19.7%) | 2620 (12%) | 12 (2.4%) |
|  | South West | 4045 (12.3%) | 1237 (11.9%) | 2758 (12.6%) | 50 (10%) |
|  | East Midlands | 964 (2.9%) | 244 (2.4%) | 714 (3.3%) | 6 (1.2%) |
|  | Yorkshire and Humber | 1086 (3.3%) | 293 (2.8%) | 792 (3.6%) | <5† |
|  | North East | 960 (2.9%) | 109 (1.1%) | 808 (3.7%) | 43 (8.6%) |

|  | North West | 5996 (18.3%) | 2207 (21.3%) | 3742 (17.1%) | 47 (9.4%) |
| --- | --- | --- | --- | --- | --- |
|  | West Midlands | 6475 (19.8%) | 1524 (14.7%) | 4976 (21.9%) | 155 (30.9%) |
|  | South Central | 3795 (11.6%) | 955 (9.2%) | 2767 (12.6%) | 73 (14.5%) |
| Ethnicity | White | 26720 (81.5%) | 8386 (80.9%) | 17894  (81.7%) | 440 (87.6%) |
|  | Mixed | 65 (0.2%) | 17 (0.2%) | 47 (0.2%) | <5† |
|  | Asian or Asian British | 454 (1.4%) | 157 (1.5%) | 284 (1.3%) | 13 (2.6%) |
|  | Black or Black British | 1175 (3.6%) | 461 (4.4%) | 706 (3.2%) | 8 (1.6%) |
|  | Chinese or Other Group | 36 (0.1%) | 12 (0.1%) | 23 (0.1%) | <5† |
|  | Number of patients with missing data | 4327 (13.2%) | 1332 (12.9%) | 2956 (13.5%) | 39 (7.8%) |
| Index of multiple  deprivation quintile | Number of patients with available data | 32745 (99.9%) | 10349  (99.9%) | 21895  (99.9%) | 501 (99.8%) |
|  | 1 (least deprived) | 8222 (25.1%) | 2587 (25%) | 5507 (25.1%) | 128 (25.5%) |
|  | 2 | 7620 (23.3%) | 2484 (24%) | 5018 (22.9%) | 118 (23.5%) |
|  | 3 | 6597 (20.1%) | 2136 (20.6%) | 4355 (19.9%) | 106 (21.1%) |
|  | 4 | 5550 (16.9%) | 1784 (17.2%) | 3675 (16.8%) | 91 (18.1%) |
|  | 5 (most deprived) | 4756 (14.5%) | 1358 (13.1%) | 3340 (15.2%) | 58 (11.6%) |
|  | Number of patients with missing data | 32 (0.1%) | 16 (0.2%) | 15 (0.1%) | <5† |

2

3 Definitions: LHRH=Luteinising hormone-releasing hormone; Q1-Q3=First Quartile-Third Quartile.
4 * Followed from date of first prescription until earliest event of death, transfer out of general
5 practice, last record collection from general practice no further Hospital Episode Statistics entry, or
6 365 days with no LHRH agonist injection prescription post-date of missed injection.
7 ** As data are drawn from medical records, the absence of a comorbidity implies the condition is not
8 present rather than data not being available. As the study population have PC, a condition included
9 in the CCI score no patients can have a score of zero
10 † When reporting the data, CPRD policy is that no cell should contain <5 events 11

12

13

14

15

16

17

18

19

20

21

22

23

24

25

26

27

28

29

30

32	*Calculations based on 2011 Office of National Statistics data for males > 40 years in England 33

34

35

36

37

38

39

40

41

42

43

44

45

46

47

48

49

50

51

| Metrics | | Whole study population | 1-monthly LHRH agonist cohort | 3-monthly LHRH agonist cohort | 6-monthly LHRH agonist cohort |
| --- | --- | --- | --- | --- | --- |
| Proportion of days covered (PDC) | Mean | 0.91 | 0.90 | 0.91 | 0.91 |
|  | Standard deviation | 0.11 | 0.13 | 0.10 | 0.08 |
|  | Median | 0.94 | 0.94 | 0.94 | 0.94 |
|  | Maximum | 1.00 | 1.00 | 1.00 | 1.00 |
|  | Minimum | 0.16 | 0.16 | 0.23 | 0.46 |
|  | Q1-Q3 | 0.87 – 0.98 | 0.86 – 0.99 | 0.87 – 0.98 | 0.9 – 0.96 |
| PDC by intervals | <80% | 4288 (13.1%) | 1653 (16.0%) | 2584 (11.8%) | 51 (10.2%) |
|  | 80% - <85% | 2559 (7.8%) | 813 (7.8%) | 1720 (7.9%) | 26 (5.2%) |
|  | 85% - <90% | 4103 (12.5%) | 1297 (12.5%) | 2748 (12.5%) | 58 (11.6%) |
|  | 90% - <95% | 6888 (21.0%) | 1885 (18.2%) | 4799 (21.9%) | 204 (40.6%) |
|  | 95% -  <100% | 10453 (31.9%) | 2798 (27.0%) | 7522 (34.3%) | 133 (26.5%) |
|  | 100% | 4486 (13.7%) | 1919 (18.5%) | 2537 (11.6%) | 30 (6.0%) |
| Total number of injections | | 368,803 | 128,033 | 238,232 | 2,538 |

53 Definitions: LHRH=Luteinising hormone-releasing hormone; PDC=Proportion of days covered; Q1-
54 Q3=First Quartile-Third Quartile

56

57

58

59

60

61

62

63

64

65

66

67

68

69

70

71

72

73

74

75

76

77

78

79

80

81

82

83

84

85

| 86 | **Figure 2. Study population flow chart** |  | |
| --- | --- | --- | --- |
| 87 |  |  |  |
| 88 |  |  |  |
| 89 |  |  |  |
| 90 |  |  |  |
| 91 |  |  |  |
| 92 |  |  |  |
| 93 |  |  |  |
| 94 |  |  |  |
| 95 |  |  |  |
| 96 |  |  |  |
| 97 |  | <5* | <5* |
| 98 |  |  |  |
| 99 | * When reporting the data, CPRD policy is that no cell should contain <5 events |  |  |
| 100 |  |  |  |
| 101 |  |  |  |
| 102 |  |  |  |
| 103 |  |  |  |
| 104 |  |  |  |
| 105 |  |  |  |
| 106 |  |  |  |
| 107 |  |  |  |
| 108 |  |  |  |
| 109 |  |  |  |
| 110 |  |  |  |
| 111 |  |  |  |
| 112 |  |  |  |
| 113 |  |  |  |
| 114 |  |  |  |
| 115 |  |  |  |
| 116 |  |  |  |

117

118

119

120

121

122

123

**Figure 3. Patient adherence measured by delayed LHRH agonist injection statistics**

124

125

126	**Figure 4. Kaplan Meier curves for the overall survival estimates for each LHRH agonist formulation,**

127	**further stratified for PDC coverage**

128

129

130	**Figure 5. Graph showing the primary causes of death in descending order for the top six conditions**

131

132

133

134	Definitions: CIHD=chronic ischaemic heart disease; MI=myocardial infarction; MN=malignant neoplasm 135

136

137

138

139

140

141

142

143

144

145

146

147

**Figure 6. Graph showing the secondary causes of death in descending order for the top 6 conditions**

148

149

150

Definitions: CIHD=chronic ischaemic heart disease; COPD=chronic obstructive pulmonary disease
