# Supplementary material for: A retrospective cohort study assessing medication coverage in patients with prostate cancer prescribed luteinizing hormone releasing hormone (LHRH) agonists in England
Source: PLoS One. 2025 Mar 21;20(3):e0315208. doi: 10.1371/journal.pone.0315208 (PMC11927884; doi:10.1371/journal.pone.0315208)
Supplement: Attachment — Submitted filename: Response to Reviewers 151124.docx [file pone.0315208.s003.docx]

**Dear PLOS ONE,**

Thank you for the opportunity to revise our manuscript, [PONE-D-24-14050]. We would like to express our appreciation to the reviewers and the editorial team for their insightful comments and suggestions. We have carefully addressed each of the points raised, and we believe the manuscript has improved as a result. Below, we provide a detailed, point-by-point response to each of the reviewer’s comments.

**Response to Reviewer Comments:**

**1. Is there any inclusion and exclusion criteria about inj LHRH agonist 1, 3, and 6 months?**

- **Response**: We appreciate the reviewer's comment. The inclusion criteria had already been mentioned in the methodology section. In response to this comment, we have now added the exclusion criteria as well, which can be found in the revised version of the methodology section.
  **Location in manuscript**: Line 169.

**2. Is there any reasoning about why the injections are delayed? (Perhaps based on patients’ condition, healthcare system, or anything else?)**

- **Response**: The methodology section of the manuscript does not originally address reasons for delayed injections. However, we have explored this issue in greater depth in the discussion section of the paper, where we have added a detailed examination of potential reasons for delays, including patient condition and healthcare system factors.
  **Location in manuscript**: Line 365.

**3. Does patients' comorbidity affect the delayed injections?**

- **Response**: We have expanded the discussion to now include an explanation of how comorbidities can influence the timing of LHRH agonist injections. This additional detail should clarify the potential role of comorbid conditions in contributing to injection delays.
  **Location in manuscript**: Line 375.

**4. How to determine prostate cancer as a primary cause of death if patients also have comorbidities?**

- **Response**: The primary cause of death is determined by the death certificate, which serves as the definitive and legally binding document used for clinical research. As such, in this study, the cause of death was based on the death certificate as the most reliable source of information.
  **Location in manuscript**: No specific reference, general clarification.

**Additional Revisions and Compliance with PLOS ONE Guidelines:**

1. **Ensured adherence to PLOS ONE style requirements**:
   We have thoroughly reviewed and revised the manuscript to ensure full compliance with PLOS ONE style requirements, including formatting and citation guidelines.
2. **Amended the role of funder statement**:
   We have revised the role of the funder statement, which is now included appropriately in the manuscript.
3. **Amended the funding statement**:
   The funding statement has been updated to declare the commercial affiliation of Ipsen authors as per PLOS ONE’s requirements.
4. **Updated competing interest statement**:
   We have amended the competing interest statement to clarify that "This does not alter our adherence to PLOS ONE policies on sharing data and materials."

**Data Sharing Request Response:**

- **Response to request for raw data**:

We have reviewed previous PLOS ONE publications that use data from CPRD and HES. Our data sharing statement has been updated to align with CPRD’s requirements and is consistent with the guidelines followed by previously accepted manuscripts:

Data are available upon request from the Clinical Practice Research Datalink (CPRD). However, access to this data requires the purchase of a license, and the terms of this license do not permit the authors to make the data publicly available to all. Licenses are available directly from CPRD (<http://www.cprd.com>): The Clinical Practice Research Datalink Group, The Medicines and Healthcare products Regulatory Agency, 10 South Colonnade, Canary Wharf, London E14 4PU.

**The data utilised in this research are classified as third-party data, meaning they are not owned or directly collected by the authors. It is confirmed that these data are accessible to others in the same manner as they were accessed by the authors. The authors did not possess any special access privileges that would grant them capabilities beyond those available to the general public**

**Additional Changes Made to the Manuscript:**

1. **Moved the ethics statement to the methods section**:
   As requested, the ethics statement has now been moved to the methods section of the manuscript for proper placement.
2. **Provided separate captions for each figure**:
   Each figure now has its own distinct caption, which is in accordance with PLOS ONE guidance.
3. **Tables and figures**:
   The tables have been included within the main manuscript, and all figures have been submitted separately, as specified by PLOS ONE.
4. **Reviewed reference list**:
   The reference list has been thoroughly reviewed to ensure that it is complete, correct, and fully adheres to PLOS ONE formatting requirements.

We hope that these revisions meet the expectations of the reviewers and the editorial board. We believe these changes have strengthened the manuscript, and we look forward to your feedback.

Thank you once again for your time and consideration.

Kind Regards,

Mohammad Raja
